# Colossal piezomagnetic response in magnetically pressed Zr^+4^ substituted cobalt ferrites

**DOI:** 10.1038/s41598-017-08160-1

**Published:** 2017-08-11

**Authors:** Monaji Vinitha Reddy, Abdellah Lisfi, Sabin Pokharel, Dibakar Das

**Affiliations:** 1 School of Engineering Sciences and Technology, University of Hyderabad, Hyderabad, AP 500046 India; 2 Department of Physics, Morgan State University, Baltimore, MD 21251 USA

## Abstract

A remarkable 111% increase in magnetostriction (λ) and 435% increase in strain sensitivity (d*λ*/d*H*) (compared to normally compacted (NC) unsubstituted CoFe_2_O_4_ (CFO)) of Zr^+4^ doped CFO sample, Co_1.2_Zr_0.2_Fe_1.6_O_4_, prepared by magnetic field assisted compaction, have been reported in this study. Magnetic field assisted compaction (MC) has been employed to process Zr-doped cobalt ferrites, Co_1+x_Zr_x_Fe_2−2x_O_4_ (0 ≤ × ≤ 0.4), to further improve the magnetoelastic properties. Saturation magnetization (*M*_*S*_) and coercivity (*H*_*C*_) increase from ~426 kA/m and ~4.4 kA/m respectively, for *x* = 0, to ~552 kA/m and ~7.11 kA/m respectively for *x* = 0.2. Dramatic increase in *λ* was observed for MC samples (~ −360 ppm and ~−380 ppm for *x* = 0 and *x* = 0.2 respectively) compared to the NC samples (~−181 ppm and ~−185 ppm for *x* = 0 and *x* = 0.2 respectively). A remarkable quadruple increase in d*λ*/d*H* was observed in Zr-doped (*x* = 0.2) cobalt-ferrite (~4.3 × 10^−9^ A^−1^m) compared to that of unsubstituted cobalt-ferrite (~1.24 × 10^−9^ A^−1^m), while a fivefold increase in d*λ*/d*H* was observed for magnetically compacted (MC) Zr doped cobalt ferrite (*x* = 0.2) (~4.3 × 10^−9^ A^−1^m) compared to normal compacted (NC) unsubstituted cobalt ferrite (~0.8 × 10^−9^ A^−1^m).

## Introduction

Magnetostrictive Cobalt-ferrite (CFO), an interesting material for magnetoelectric (ME) multiferroic composites because of its high magnetostriction and strain sensitivity, has been identified as a potential material for stress or torque sensor applications^1–3^. Since the magnetostrictive property of cobalt ferrite depends on the concentration of cobalt ions (Co^+2^) in the octahedral sites of the spinel lattice, variation in synthesis methods, chemical substitution, processing techniques provide an opportunity to tailor its magnetic and magnetostrictive properties suitable for various applications^4–7^. Slonczewski and Tachiki^8, 9^ reported that at lower concentrations Co^+2^ ions in the octahedral site are surrounded by Fe^+3^ ions. These Fe^+3^ ions around the central Co^+2^ ion produce a trigonal crystal field whose axis of symmetry lies along one of the body diagonals of the spinel cubic lattice. This trigonal crystal field distorts the symmetry of the cubic octahedral field produced by the oxygen ions around Co^+2^ resulting in lifting of the degeneracy of the d-orbitals of Co^+2^ ions and produces unquenched orbital moments. Magnetostriction and anisotropy energy result from the coupling of Co^+2^ spin with its angular momentum. Hence, the environment produced by the metal cations around Co^+2^ ion in octahedral sites plays a crucial role in determining the magnetostrictive properties of cobalt ferrite. In a recent study^10^ it has been found that substitution of Zr^+4^ in CFO lattice significantly improved the maximum magnetization (M_max_), magnetostriction (λ), and strain sensitivity (dλ/dH) compared to unsubstituted CFO. Besides chemical substitution, inducing anisotropy along a particular direction is seen to enhance the magnetic and magnetoelastic properties of CFO. Kaja Mohaideen *et al*. reported^11^ a high magnetostriction and strain derivative, 380 ppm and 2.7 × 10^−9^ A^−1^m, respectively, of polycrystalline cobalt ferrite samples sintered at low temperature (1100 °C) and magnetically (magnetic field of 400 kA/m for 30 mins) annealed at 300 °C. P.N. Anantharamaiah *et al*.^12^ reported a high strain sensitivity (4.5 × 10^−9^ A^−1^m) of the magnetically annealed Al-doped cobalt ferrite sample. In a recent study by Wang *et al*.^13^ a dramatic enhancement in the strain sensitivity (2.2 × 10^−9^ A^−1^m for non-oriented sample to 7.7 × 10^−9^ A^−1^m for oriented sample) has been reported for CFO sample processed by magnetic field assisted shaping method, which is the highest value reported so far for any unsubstituted or substituted CFO sample. In the present study chemical substitution and magnetic compaction have been chosen to enhance the magnetoelastic properties of cobalt ferrite. Magnetic field assisted compaction is a well-known technique in producing anisotropic permanent magnets including hard ferrites^14^. Magnetic field applied during compaction helps in inducing better degree of alignment of domains in powders. Compaction locks the particles in that state resulting in best magnetic homogeneity in the sample^15, 16^. Compaction is an important step in the processing of CFO by conventional solid-state method and if it is performed under the influence of magnetic field it is expected to enhance the magnetic properties. Magnetic compaction is a better technique to align the domains compared to magnetic annealing, since there is a possibility of degradation of magnetic properties of the samples particularly, when they are operated at high temperatures.

In this work, the effects of substitution of Zr^+4^ ion into the CFO lattice and the magnetic compaction (MC) on structural, magnetic, and magnetostrictive properties of cobalt ferrite have been studied and the results have been compared with those of normal compacted (NC) (compaction in the absence of any magnetic field) Zr-substituted CFO samples.

## Results and Discussion

### Structural properties

**Figure 1:**
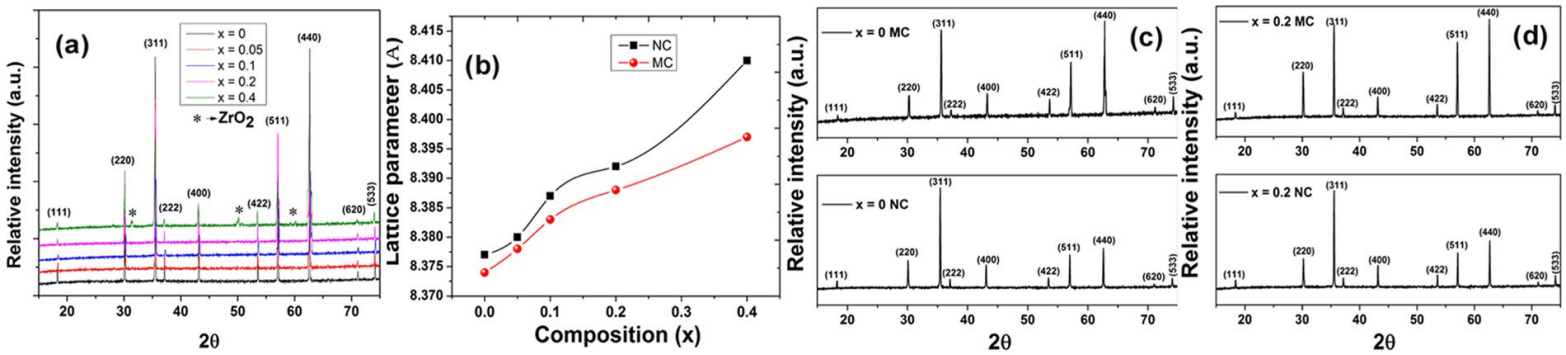
(**a**) X-ray diffraction spectra of sintered Co_1+x_Zr_x_Fe_2−2x_O_4_ (0 ≤ × ≤ 0.4)-MC samples; (**b**) The variation of lattice parameter as a function of Zr substitution, x and their comparison with normal compacted samples; (**c**) and (**d**) XRD patterns of NC and MC x = 0 and 0.2 compositions.

Figure 1(a) shows the x-ray diffraction spectra (θ/2θ scan) of the sintered pellets of Zr-substituted cobalt ferrite samples, confirming the single-phase cubic spinel structure. An overall increase in lattice parameter (from ~8.374 Å for x = 0 to ~8.397 Å for x = 0.4), as shown in Fig. 1(b), has been observed with progressive Zr substitution in the cobalt ferrite lattice. CoFe_2_O_4_ has partially inverse spinel structure and the inversion factor, which depends on the synthesis method and annealing temperature, is expected to affect the lattice parameter. O’Neill and Navrotsky reported^17^ the calculated lattice parameters of (Co^+2^)[Fe^+3^]O_4_ and (Fe^+3^)[Co^+2^Fe^+3^]O_4_ as 8.4091 Å and 8.3702 Å, respectively. Sawatzky *et al*.^18^ estimated the cation distribution of CoFe_2_O_4_ from MÖssbauer spectroscopy and reported it as (Co_0.3_^+2^Fe_0.7_^+3^)[Co_0.7_^+2^Fe_1.3_^+3^]O_4_. The calculated lattice parameter of this distribution (~8.3826 Å) is in excellent agreement with the value reported by O'Neill and Navrotsky. Since Zr^+4^ ions have strong preference for tetrahedral coordination, they prefer to occupy the tetrahedral (A) sites of the cubic spinel lattice^19^. The observed increase in lattice parameter of Co_1+x_Zr_x_Fe_2−2x_O_4_ (0 ≤ × ≤ 0.4) samples could be due to the fact that the ionic radii of Zr^+4^ and Co^+2^ in tetrahedral and octahedral co-ordination, respectively, are greater than those of Fe^+3^ ions with similar coordination number. Based on the calculated lattice parameters reported in ref. 17, the increasing non-linearity in lattice parameter after x = 0.1 for NC sample could be due to the occupation of more Co^+2^ ions in the tetrahedral sites of spinel lattice along with Zr^+4^ ions.

From the comparison of XRD patterns of magnetic field and normal pressed x = 0 and x = 0.2 samples, as shown in Fig. 1(c) and (d), respectively, increase in intensities of the Bragg reflections are observed for (220), (511) and (440) planes in the magnetic field pressed sample, which indicates the formation of texture along those planes. The effect of texture on magnetostriction will be discussed in the respective section.

### Microstructural properties

**Figure 2:**
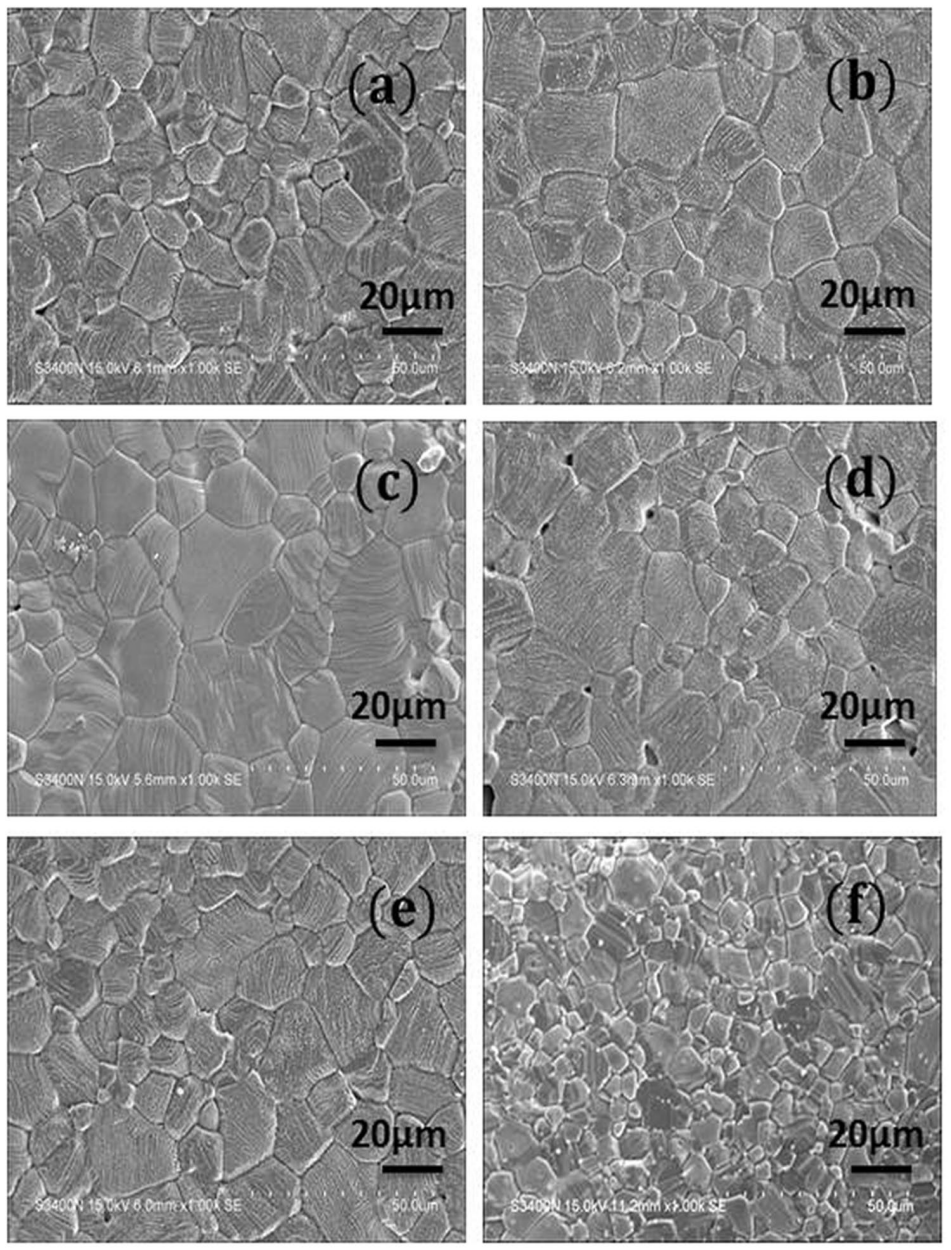
(**a**,**b**) Scanning electron micrographs of the NC and MC sintered cobalt ferrite samples respectively; (**c**–**f**) Scanning electron micrographs of sintered pellets of x = 0.05, 0.1, 0.2, 0.4 samples, respectively.

The SEM images of the sintered Zr substituted cobalt ferrite (Co_1+x_Zr_x_Fe_2−2x_O_4_ (0.0 ≤ × ≤ 0.4)) samples are shown in Fig. 2. Figure 2(a) and (b) represent the microstructures of normally and magnetically pressed (NC and MC respectively) unsubstituted cobalt ferrite samples. The average grain sizes, as estimated from the line intercept method, have been found to be around 10 and 20 μm for CFO-NC and CFO-MC samples, respectively. In general, in the compacted samples some preferred orientation should take place at right angles to the direction of compaction^15^. In case of samples compacted in presence of magnetic field, along with the effect of compaction, domains in the particles get aligned in the direction of the magnetic field. Because of the high agglomeration of single domain particles during the pressing operation high green density was achieved, which resulted in high sintered density and increased grain growth compared to those obtained from normal compacted samples. With increasing Zr^+4^ concentrations not much difference in the grain size was observed, except for x = 0.4. The observed decrease in grain size for x = 0.4 could be due to the presence of extra ZrO_2_ phase in the grain boundaries, which impedes the grain growth.

### Magnetic properties

#### Magnetization

**Figure 3:**
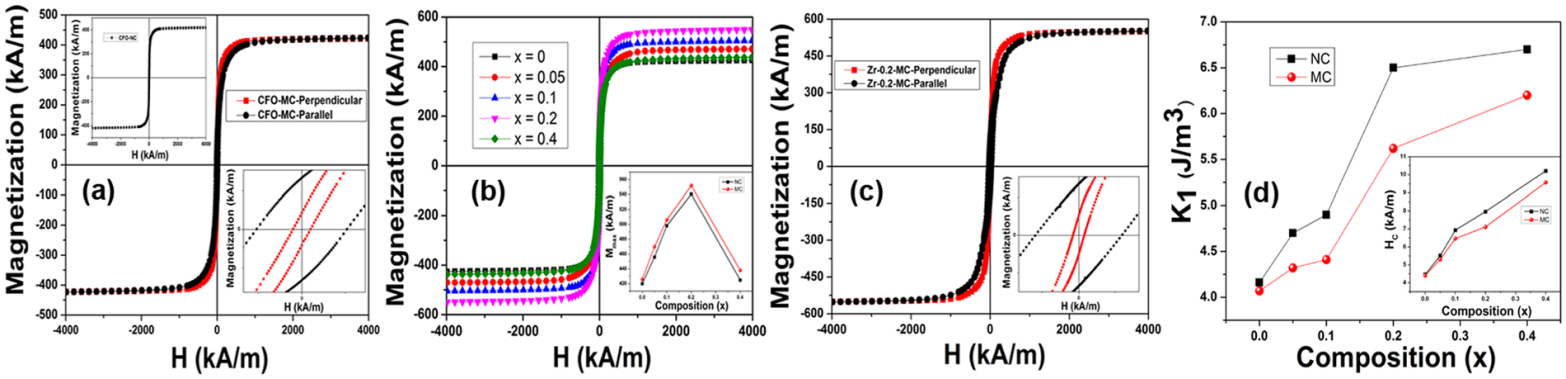
(**a**) Parallel and perpendicular M-H curves of x = 0, inset of (**a**) shows magnetization curve of CFO-NC; (**b**) Magnetization (M-H) curves of Co_1+x_Zr_x_Fe_2−2x_O_4_ (0 ≤ ×≤ 0.4)-MC samples at room temperature (~300 K); (inset) Variation of M_S_ with composition (x); (**c**) Parallel and perpendicular M-H curves of x = 0.2 composition, (**d**) Variation of anisotropy constant and coercivity (inset) as a function of composition and its comparison with NC samples.

**Figure 4:**
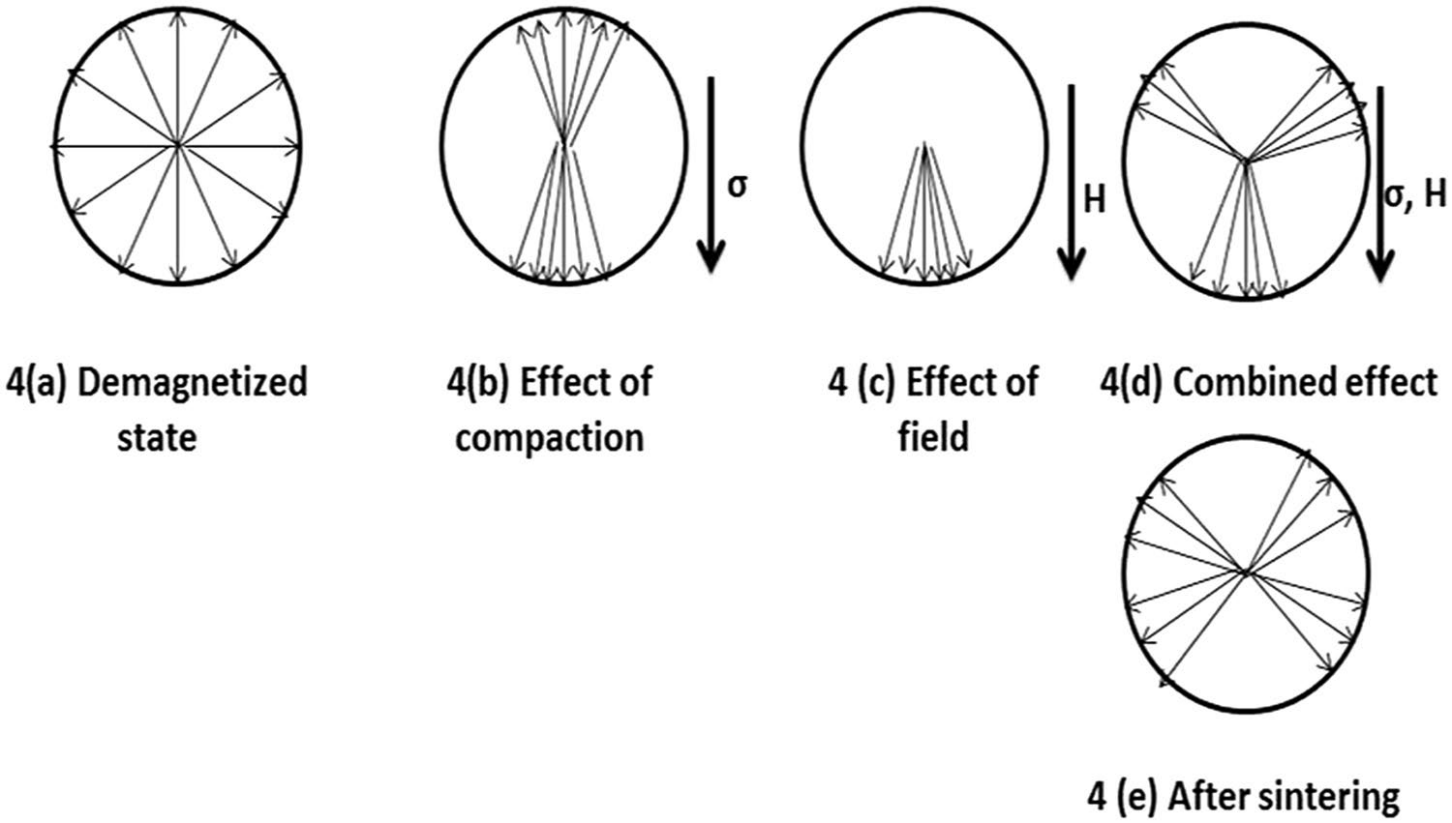
A schematic of domain alignment during magnetic compaction.

Figure 3 shows the hysteresis loops of normally and magnetically pressed CoFe_2_O_4_ samples. Qualitative descriptions of domain alignment during magnetic pressing are shown schematically in Fig. 4. Direction of magnetization in a domain depends on the crystal structure when the external stress and magnetic field are absent and these are the two main controlling factors to change the domain orientation. When the sample is compacted in the presence of magnetic field, magnetic strain energy (Eσ) and domain energy in the presence of applied magnetic field (E_H_) come into picture and these energies are given by the following relations^20^,

1 $${E}_{\sigma }=\frac{3}{2}{\lambda }_{s}\,\sigma \,{\sin }^{2}\theta $$

2 $${E}_{H}=-H\,{M}_{s}\,\cos \,\varphi $$

where λs, σ, H and Ms are the saturation magnetostriction, applied stress, field strength and saturation magnetization, respectively. Angles θ and ϕ represent orientation of magnetization in a domain with respect to stress and applied field, respectively. During compaction (compressive stress) all the domains in the powder particles are oriented nearly to the direction of applied stress (θ = 0 or 180°) due to the two-fold symmetry of the uniaxial anisotropy generated by compaction. More precisely, the preferential direction of the magnetization will be close to the stress axis due to the negative magnetostriction (λs) of cobalt ferrite and compressive stress (‘σ’ negative) applied during compaction. Figure 4(a) represents the ideal demagnetized state. To align the domains, compressive stress must be large enough compared to the forces due to crystal anisotropy, therefore from both the energy considerations the condition to orient the magnetization direction in a domain is

3 $$\frac{3}{2}{\lambda }_{s}\sigma  > {K}_{1}$$

For cobalt ferrite the first order anisotropy constant K_1_ is about 4 × 10^5^ J/m^3^. The energy of the applied stress (left side quantity in Eq. 3) in the present investigation is greater than the crystal anisotropy; hence it orients the domain in the direction of compaction as shown in Fig. 4(b) due to the two-fold symmetry of the stress field energy (Eq. 1). Applied magnetic field during compaction tries to orient the domains in the field direction as shown in 4(c) due to the one-fold symmetry of the Zeeman energy generated by the magnetic field (Eq. 2). The resultant effect of these two energies orients the domains in the green compact as shown in 4(d). During sintering at 1300 °C, which is far greater than the Curie temperature (520 °C) of cobalt ferrite spin orientations may get randomized, but due to strong lattice-orbit coupling the resultant magnetization vectors align along the direction near to the initial stable magnetization direction after cooling, which is shown in Fig. 4(e). Here the perpendicular direction to the direction of compaction can be considered as induced easy axis or first easy axis. Intrinsic easy axes for cobalt ferrite material are <100> axes, hence the <100> axes near 90° to the induced easy axes can be considered as second easy axes.

From the M-H loop of CFO - NC, which is shown in the inset of Fig. 3(a) (left side top corner), it is observed that, initially with increasing applied magnetic field, alignment of domains takes place along the intrinsic easy axes through 180 and 90° domain wall motion if they are initially oriented at small and large angles, respectively, to the applied field direction. As the applied magnetic field further increases magnetization vector rotates along the field direction. The measured M-H loops of CFO-MC sample with the measuring field applied parallel and perpendicular to the compaction direction are shown in the main panel of Fig. 3(a). From the perpendicular magnetization data of CFO-MC, it is observed that with increasing magnetic field magnetization increases more quickly, which could be due to the initial alignment of most of the domains along the induced easy axes during magnetic compaction. Here, when the magnetic field increases domains get aligned along the induced easy axes only through 180° domain wall motion. In contrast, when magnetization of CFO-MC was measured in the parallel direction to that of pressing direction, magnetization process can be explained in three stages. In the first stage, with increasing magnetic field domains get oriented initially along the induced easy axes through 180° domain wall motion. In the second stage, further increment in magnetic field strength leads to a near 90° domain wall motion, which aligns the domains along the second easy axes (intrinsic easy axes). In the third stage, with increasing magnetic field magnetization of domains gradually rotates from the second easy direction to the direction of applied magnetic field. Increment in magnetization in all these stages is small compared to perpendicular magnetization measurement, because initially the domains are oriented at large angles to the direction of applied field. Inset of Fig. 3(a) (right side down corner) will be discussed in the coercivity section of this manuscript.

Figure 3(b) shows the field dependent perpendicular magnetization data of magnetically pressed Co_1+x_Zr_x_Fe_2−2x_O_4_ (0 ≤ × ≤ 0.4) samples measured at room temperature. The maximum magnetization (M_max_) obtained at maximum applied field of ~4000 kA/m is seen to increase marginally with increasing Zr substitution up to x = 0.2. Further increase in Zr content resulted in decreasing magnetization primarily due to the presence of extra ZrO_2_ phase at the grain boundaries. In spinel structure, super-exchange interaction between the cations in tetrahedral (A) and octahedral (B) sites is generally anti parallel. Therefore, the overall magnetization is the difference in the magnetization of the A and B sub lattices. In general, Co-ferrite has mixed spinel structure and the inversion factor mainly depends on the synthesis methods, chemical substitution and annealing, since these processes redistribute the cations between the A- and B-sites of the spinel lattice. In case of Co_1+x_Zr_x_Fe_2−2x_O_4_ (0 ≤ × ≤ 0.2), substitution of non-magnetic Zr^+4^ ions reduces the magnetic moment of tetrahedral sub lattice, since Zr^+4^ ions have strong preference for tetrahedral site, which resulted in increased net magnetization of the system. Inset of Fig. 3(b) shows the comparison of maximum magnetization of magnetically pressed (MC) to that of normally pressed (NC) Zr- substituted cobalt ferrites. Marginal increment in magnetization is observed in the MC samples, which could be due to the presence of more aligned domains structure. Since the magnetization is increased up to x = 0.2, to see the effect of magnetic pressing on Zr doped sample parallel magnetization measurement was also carried out on x = 0.2 composition. Figure 3(c) shows the parallel and perpendicular M-H loops of Co_1.2_Zr_0.2_Fe_1.6_O_4_ sample. From this figure it is clearly observed that perpendicular magnetization curve is steeper than the parallel magnetization curve. Substitution of non-magnetic ion into the CFO-lattice reduces the strength of magnetic exchange interaction, which helps in aligning the domains easily along the direction of induced easy axis and thus should influence the magnetization behavior, which is clearly reflected in Fig. 3(c). Inset of Fig. 3(c) will be discussed in the coercivity section of this manuscript.

#### Anisotropy constant estimated using law of approach to saturation

It has already been reported that the cubic anisotropy constant of cobalt ferrite mainly depends on the concentration of Co^+2^ ions in the B-sites of spinel structure. Due to the cubic crystal field produced by the coulomb interaction between the oxygen ion and the electrons in the 3d orbitals, degenerate energy levels of Co^+2^ ion are split into t_2g_ (d_xy_, d_yz_, d_xz_) and e_g_ (d_x_^2^_−y_^2^, d_z_^2^) levels and the orbitals, which are situated along the cubic axes (e_g_ levels) are raised in energy compared to others (t_2g_ levels). For lower concentrations of Co^+2^ ions in B site, neighboring iron ions produce trigonal field around Co^+2^ ion and the axis of three fold symmetry lies along one of the body diagonals ([111], [1–11], [−1–11], [−111]) of the cubic crystal. This trigonal crystal field splits the t_2g_ levels further into singlet and a doublet by distorting the cubic crystal field produced by the oxygen ions. Occupation of seventh electron of Co^+2^ (3d^7^) in one of these doubly degenerate levels produces unquenched orbital momentum and interaction of this orbital angular momentum with total spin momentum of Co^+2^ ion gives rise to magneto crystalline anisotropy through spin-orbit coupling. In the previous study it is reported that the orbital degeneracy is further removed when the Co^+2^ concentration in B-site increases beyond × >0.7 due to the low symmetric crystalline field produced from the difference in charge between two metal cations (Co^+2^ and Fe^+3^), which leads to quenching of the orbital angular momentum and subsequently reduces magnetocrystalline anisotropy. Hence, any variation in concentration of Co^+2^ ions in B-site results in change in the magnetocrystalline anisotropy of the system.

Magnetization processes in the high field region are predominantly due to the domain rotation against anisotropy energy. Hence, the cubic anisotropy constant (K_1_) can be estimated using the “Law of Approach” to saturation, which describes the relation between magnetization and applied field in the saturation region as,

4 $$M={M}_{s}(1-\frac{b}{{H}^{2}}-\,---)+\kappa H$$

here ‘κH’ represents the forced magnetization and it can be ignored if the measurement is performed at temperature far below the curie temperature. M_S_ and H are the saturation magnetization and applied field, respectively. The constant ‘b’ is related to the crystal anisotropy of the system and for randomly oriented polycrystalline samples it can be interpreted as,

5 $$b=(\frac{8}{105})(\frac{{{K}_{1}}^{2}}{{{\mu }_{0}}^{2}\quad {{M}_{s}}^{2}})$$

The variation in anisotropy coefficient (K_1_), estimated by fitting the magnetization data in the high field region using Eq. 4, with Zr content is shown in Fig. 3(d). The anisotropy constant (K_1_) increases with increase in co-substitution of Zr^+4^/ Co^+2^, similar to the variation in coercive field (H_C_) with composition of the samples, as shown in inset of Fig. 3(d). In the present case, the increase in anisotropy constant could be attributed to the displacement of Fe^+3^ ions from A- to B-site, which enhances the symmetry of trigonal field around Co^+2^ ion in B-site, thus increases the spin-orbit coupling and magneto crystalline anisotropy. Figure 3(d) also depicts that for all the magnetically pressed Co_1+x_Zr_x_Fe_2−2x_O_4_ (0 ≤ × ≤ 0.4) samples anisotropy constant decreased compared to normally pressed samples, which might be due to the induced anisotropy along the first easy axis. In general, energy associated with the spin-orbit coupling at B-sites, which lie on the trigonal axis ([111], [1–11], [−1–11], [−111]) is equal, hence there is an equal probability for Co^+2^ ions to get distributed among these four B-sites. But, when the magnetic field is applied during compaction, resultant magnetization vector of spin-orbit interaction prefers to orient along the induced easy axis. Therefore, during heat treatment there is a probability of migration of Co^+2^ ions to the B-sites with its trigonal axes, which are nearer to induced easy axis and hence resulting in induced anisotropy.

#### Torque curves

**Figure 5:**
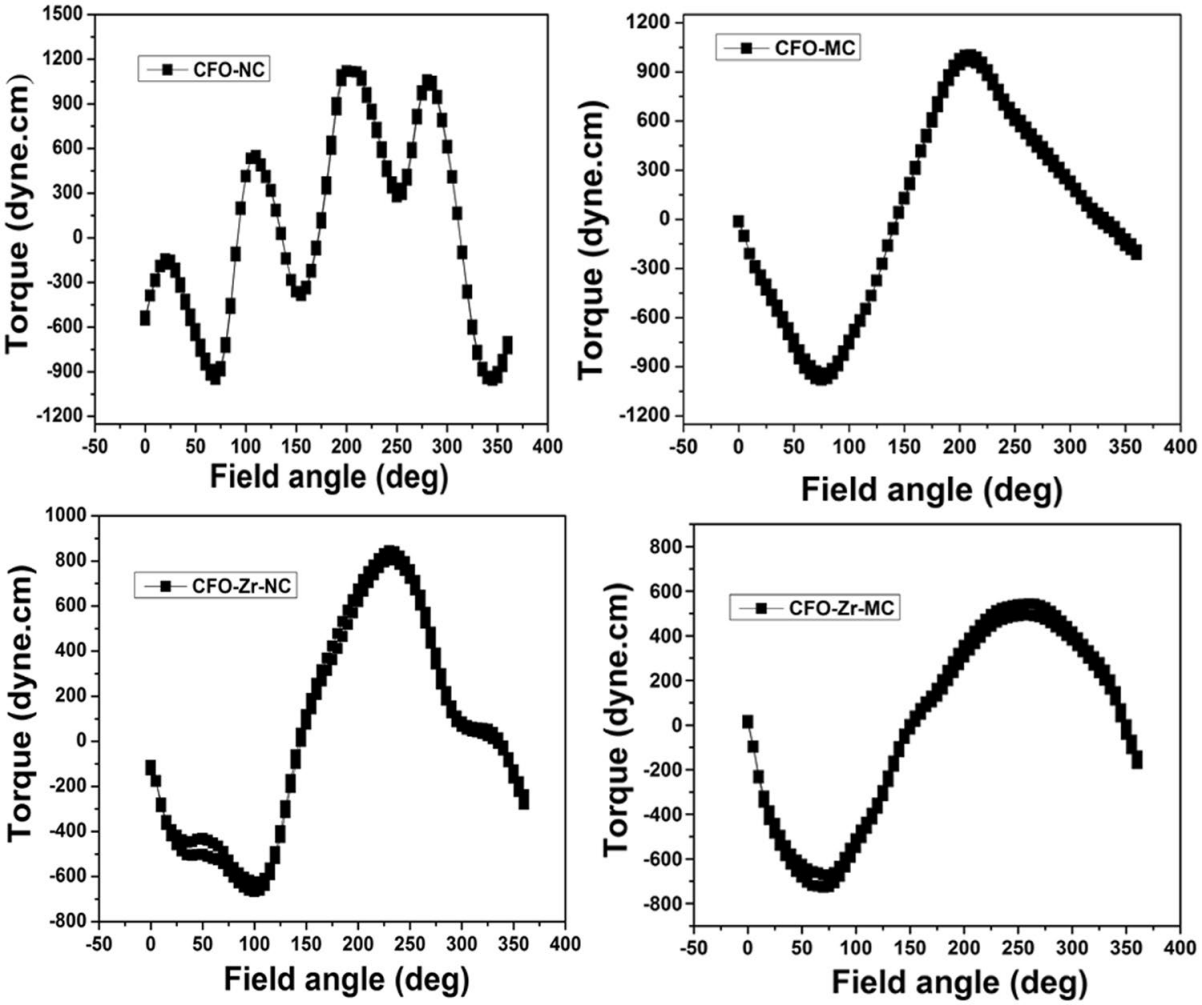
Torque curves of (**a**) x = 0 (**b**) x = 0.2 NC and MC samples.

The torque measurement is an important technique to estimate the anisotropy constant. Torque curves of Co_1+x_Zr_x_Fe_2−2x_O_4_ (x = 0 and 0.2) normally and magnetically pressed poly crystalline samples are shown in Fig. 5(a) and (b). The figures clearly depict that the measured torque curves of both the systems are not periodic; hence Fourier analysis cannot be used to estimate the anisotropy constant. Fourier analysis requires oriented magnetic anisotropy for which the periodic torque curve can be decomposed in different harmonics with coefficients, which basically represent the different orders (first, second...) of magnetic anisotropy. For an oriented magnetic system, the torque is zero when the field is applied parallel to the easy or the hard axis of magnetization and the two axes can be differentiated by the slope of the torque curve (the slope is negative at easy axis and positive at hard axis). For non-oriented (isotropic) magnetic system, since all the directions are equivalent the torque is zero. But for the magnetic material with different textures, the torque will be the superposition of each texture. In such type of materials zero torque at a particular angle indicates that the net torque is zero due to the contribution from each texture. From Fig. 5(a) and (b) it is clearly observed that the samples are exhibiting non-zero torque; it implies that the grains are having some texture with random anisotropy. Therefore, the only way to measure the anisotropy constant of polycrystalline materials is by analyzing the rotational hysteresis from the field dependent torque measurements. The anisotropy field (H_a_) at which the reversible torque curve without hysteresis is produced could be used to estimate the anisotropy constant from the following formula,

6 $${{\rm{H}}}_{{\rm{a}}}=\,\frac{2{{\rm{K}}}_{1}}{{{\rm{M}}}_{{\rm{s}}}}$$

where K_1_ is the anisotropy constant and M_s_ is the saturation magnetization. Even though the values of anisotropy constants of NC and MC samples estimated from Eq. 6, as shown in Table 1, are different from those estimated from law of approach to saturation, they are following the same trend. The different jumps at different angles observed in the Fig. 5(a) and (b) indicate the orientation of grains in different directions and the jumps are observed to reduce in the magnetically compacted samples compared to the normally compacted ferrite samples. Therefore, it indicates that more aligned domains are present in the magnetic pressed samples.

**Table 1:** Comparison of anisotropy constant estimated from law of approach to saturation and from torque curves.

Composition	K_1_ = (105/8) (bµ_0_^2^M_S_^2^) (× 10^5^ J/m^3^)	K_1_ = H_a_M_s_/2 (× 10^5^ J/m^3^)
CFO-NC	**4.16**	**2.14**
CFO-MC	**4.07**	**2.09**
CFO-ZR-0.2-NC	**6.5**	**5.52**
CFO-ZR-0.2-MC	**5.62**	**5.41**

#### Coercivity

M-H loops of CFO-MC, shown in Fig. 3(a), clearly indicate that uniaxial anisotropy is induced along the perpendicular direction (induced easy axis) to that of compaction. Magnetic field induced uniaxial anisotropy during compaction reduces the magneto crystalline anisotropy. As a result, 5 times reduction in coercivity (H_c_) has been observed along the perpendicular direction compared to parallel direction as shown in the inset of Fig. 3(a) (right side down corner of Fig. 3(a)), which indicates that material has become magnetically soft along that direction. Similarly, coercivity of Co_1.2_Zr_0.2_Fe_1.6_O_4_ sample along the perpendicular direction (induced easy axis) is observed to reduce by 7 times to that of measured along the parallel direction, as shown in inset of Fig. 3(c), which could be due to the presence of large induced uniaxial anisotropy in the substituted sample compared to unsubstituted one. Dependence of coercivity (H_c_) on composition of Zr- substituted NC and MC samples is shown in inset of Fig. 3(d). Coercivity is observed to increase with increasing co-substitution of Co^+2^ and Zr^+4^ for 2 Fe^+3^ ions in the CFO lattice because it is strongly related to the anisotropy of the system, which is mainly dependent on the amount of Co^+2^ ions present in the octahedral sites. Coercivity of MC samples is observed to be smaller than the NC samples, which is mainly attributed to the presence of larger grains in the MC samples (~50 μm) compared to that of NC samples (~20 μm), since apart from anisotropy microstructure also affects the coercivity.

#### Magnetostriction

**Figure 6:**
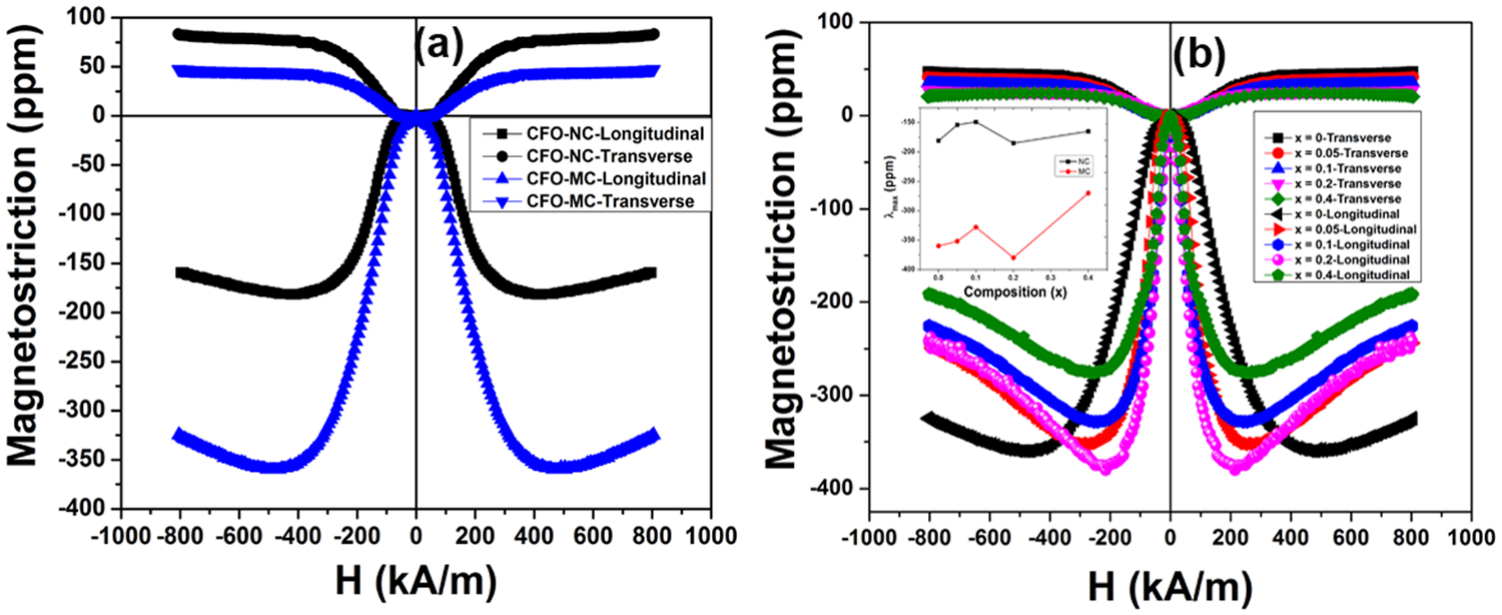
Longitudinal and Transverse magnetostriction curves of (**a**) CFO - NC and MC (**b**) Co_1+x_Zr_x_Fe_2−2x_O_4_, (0 ≤ × ≤ 0.4)-MC samples; Inset: Variation of magnitude of maximum magnetostriction as a function of composition, x and their comparison with NC samples.

The magnetostriction values of magnetically pressed Co_1+x_Zr_x_Fe_2−2x_O_4_ (0 ≤ × ≤ 0.4) samples are shown in Fig. 6. From Fig. 6(a) it is observed that for normal pressed unsubstituted CFO (CFO-NC) longitudinal magnetostriction (λ_l_) reaches the maximum of −181 ppm, when the applied magnetic field increases to ~400 kA/m and decreases marginally at higher applied magnetic field, which indicates the anisotropic nature of magnetostriction. The maximum transverse magnetostriction (λ_t_) value is approximately one half of that for the longitudinal (~88 ppm), which is in good accordance with the following relation,

7 $${\rm{\lambda }}=\frac{3}{2}{\rm{\lambda }}s\,\,({\cos }^{2}{\rm{\theta }}-\frac{1}{3})$$

where ‘θ’ is the angle between direction of magnetization and direction in which the magnetostriction is measured. θ = 0° and 90° denote the longitudinal (λ_l_) and transverse magnetostriction (λ_t_) respectively. If these two ‘θ’ values are substituted in the above equation it reduces to λ_l_ = λ_t_/2. Direction dependence of magnetostrictions of magnetically pressed unsubstituted CFO (CFO-MC) is shown in Fig. 6(a). Significant improvement in magnetostriction is observed in comparison with CFO-NC. The corresponding values of λ_l_ and λ_t_ are ~−360 ppm and ~44 ppm for the longitudinal and transverse measurements, respectively. It is interesting to note that the 2:1 relation of longitudinal and transverse magnetostriction is no longer applicable for CFO-MC sample. In this case the above Eq. (7) is no longer valid and the dependency of magnetostriction on initial domain orientation can be explained based on the following equation,

8 $${\rm{\lambda }}=\,\frac{3}{2}{{\rm{\lambda }}}_{s}( < {\cos }^{2}\,{{\rm{\theta }} > }_{{\rm{t}}}-{ < \cos }^{2}\,{{\rm{\theta }} > }_{0})$$

here <cos^2^θ>_0_ and <cos^2^θ>_t_ represent the initial domain orientation and domain orientation at any time ‘t’, respectively. Even though for a polycrystalline material it is difficult to mention the orientation direction, by relating the observed λ_l_ and λ_t_ values of CFO-MC sample with Eq. 8 it can be concluded that initially oriented domains were present in the sample along the transverse direction to that of compaction. From Fig. 6(a) it is also observed that λ_t_ of CFO-MC is smaller than that of CFO-NC. If we relate this observation with the magnetization data measured in the same direction (induced easy axis), it is seen that the magnetization increased more rapidly with the application of field due to the initial domain orientation along the induced easy axis. Even though the magnetization increases it may not contribute to dimensional changes, since the domain orientation with the applied magnetic field takes place through 180° domain wall motion. The observed small transverse magnetostriction (λ_t_) could be due to the lack of complete alignment of domains in the magnetically pressed sintered sample. Two-fold increment in longitudinal magnetostriction (λ_l_) is observed for CFO-MC sample compared to CFO-NC. This can be explained from the parallel magnetization measurement of CFO-MC sample. In this case, as explained in the previous section, with increasing applied magnetic field domains get aligned through 180° and 90° domain wall motion and domain rotation. Here 90° domain wall motion and domain rotation lead to the dimensional changes and these processes occur more in the magnetically pressed sample compared to CFO-NC, because of the presence of oriented domains along the induced easy axis.

Figure 6(b) shows the longitudinal and transverse magnetostriction (λ_l_ and λ_t_) of magnetically pressed Co_1+x_Zr_x_Fe_2−2x_O_4_ (0 ≤ × ≤ 0.4) samples. λ_l_ is seen to decrease with increasing Zr^+4^ concentration, except for x = 0.2, as shown in Fig. 6(b). Magnetostriction and cubic anisotropy of cobalt ferrite originate from spin-orbit coupling, which arises from the unquenched orbital momentum of Co^+2^ ions in octahedral site. Tachiki reported that quenching of orbital momentum of Co^+2^ ion depends on the amount of Co^+2^ ions present in the octahedral sites of spinel lattice. The observed marginal increase in magnetostriction for x = 0.2 composition compared to unsubstituted is attributable to the presence of optimal concentration of Co^+2^ ions in the octahedral site, produced from the displacement of Fe^+3^ ions from A to B site with the co-substitution of Zr^+4^/Co^+2^ ions in A-site, which is in good agreement with the variation observed in lattice parameter after x = 0.1. Decrease in magnetostriction for x = 0.4 is attributed to the presence of secondary phase (ZrO_2_) in the sample. From a careful review of the XRD data of magnetically compacted x = 0 and 0.2 samples (Fig. 1(c) and (d)) it is seen that texturing was observed along [220], [511] and [440] directions and out of these three, maximum crystal orientation was observed along [440] direction. By correlating this observation with the parallel and perpendicular data of magnetization and magnetostriction it can be inferred that [440] direction could be the induced easy axis in the magnetically compacted samples. From the quantitative analysis of domain alignment, as discussed in magnetization section, it has been concluded that the perpendicular direction (to that of pressing direction) could be the induced easy axis. Therefore, the direction which is perpendicular to the [440] direction could be the pressing direction. Magnetically pressed Co_1+x_Zr_x_Fe_2−2x_O_4_ (0 ≤ × ≤ 0.4) samples have shown large negative magnetostriction values, therefore [001] direction might be the pressing direction, since it is perpendicular to [440] direction and is one of the easy axes (<100>) of cobalt ferrite, along which CoFe_2_O_4_ has well known negative magnetostriction. The maximum magnetostriction values observed at lower fields could be due to the low angle alignment of [100] direction (one of the easy axes of cobalt ferrite) to [511] and [440] directions. Inset of Fig. 6(b) depicts the variation in magnetostriction of normally and magnetically pressed Co_1+x_Zr_x_Fe_2−2x_O_4_ (0 ≤ × ≤ 0.4) samples. Approximately 111% increment in longitudinal magnetostriction is observed for magnetically pressed Co_1.2_Zr_0.2_Fe_1.6_O_4_ sample compared to CFO-NC. Small decrease in λ_t_ is observed with increasing Zr^+4^ concentration into the spinel lattice.

#### Strain sensitivity

**Figure 7:**
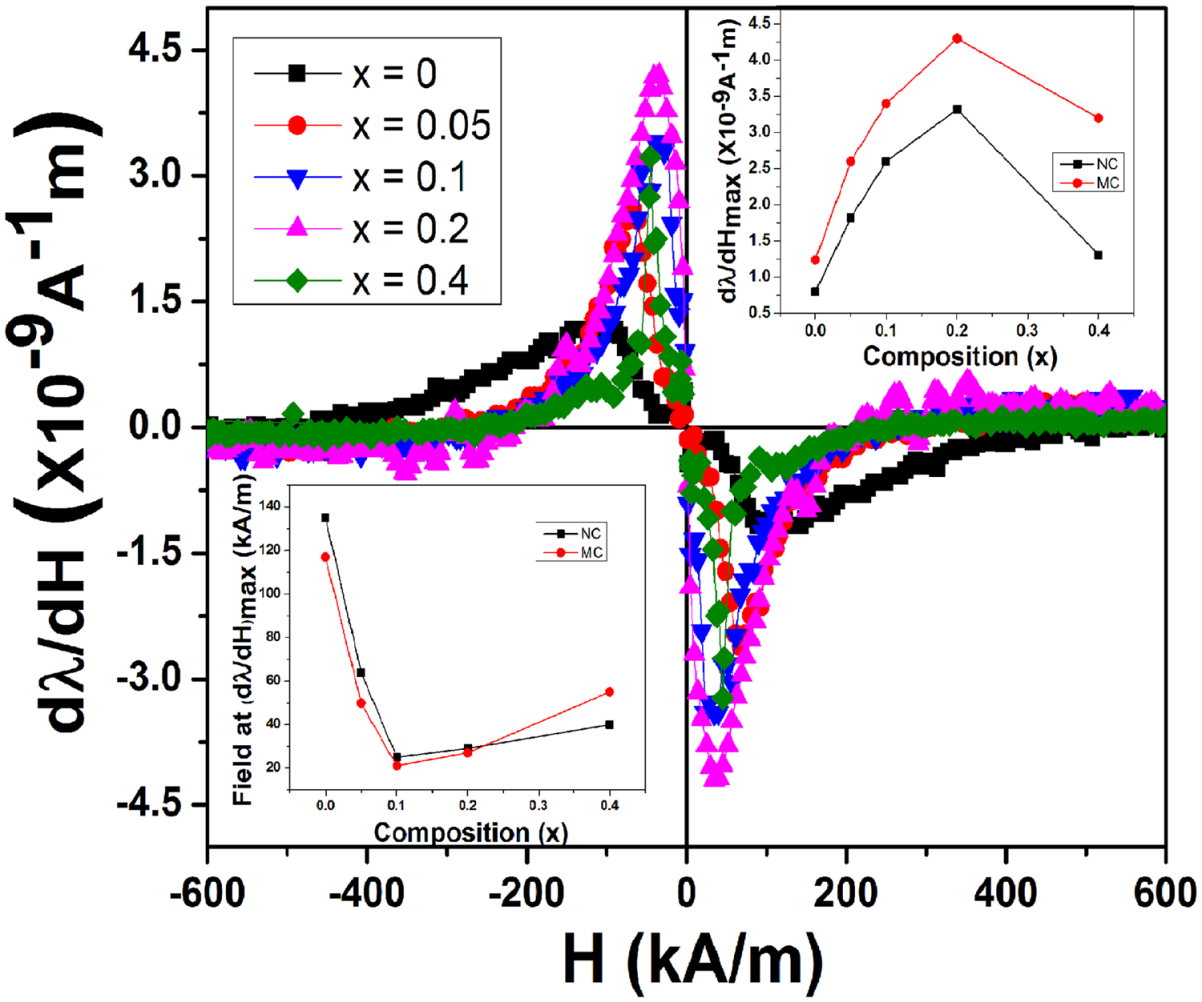
Magnetic field (H) dependence of strain derivative (dλ/dH) for Co_1+x_Zr_x_Fe_2−2x_O_4_, (0 ≤ × ≤ 0.4)-MC samples. Inset: Variation of maximum strain derivative, (dλ/dH)_max_ (Top right corner) and field (H) required to achieve that (lower left corner) as a function of composition (x).

Strain sensitivity (dλ_l_/dH) of magnetically compacted Co_1+x_Zr_x_Fe_2−2x_O_4_ (0 ≤ × ≤ 0.4) samples, as a function of applied magnetic field, is shown in Fig. 7. Progressive Zr^+4^ ion substitution into the CFO lattice resulted in increasing (dλ_l_/dH)_max_ up to x = 0.2 composition, which is ascribed to the reduced super exchange interaction between tetra and octahedral sub-lattices in the presence of non-magnetic Zr^+4^ ion in the spinel lattice. For x = 0.4, (dλ_l_/dH)_max_ is observed to decrease due to the hindrances produced by the extra phase to the domain wall motion and also might be due to the magnetic dilution induced by the presence of more amount of Zr^+4^ in the spinel lattice. Effective magnetostriction at lower fields results from the domain orientation through non-180° domain wall motion and as explained earlier these processes occur more quickly in magnetically compacted samples due to the initial orientation of domains along first (induced) easy axis. Hence, large enhancement in strain derivative was observed for magnetically pressed Zr-substituted cobalt ferrites compared to normally pressed samples. The effect observed at lower fields in magnetization (sensitivity of magnetization (dM/dH)) and magnetostriction (strain sensitivity (dλ/dH)) curves of NC and MC samples is shown in Fig. S1 in the supplementary section, where the correlation between strain sensitivity and sensitivity of magnetization is obvious. Inset of Fig. 7 (top right corner) shows the comparison of (dλ_l_/dH)_max_ of magnetically and normally pressed samples as a function of composition. From this figure ~55% increment in (dλ_l_/dH)_max_ is observed for CFO-MC sample (1.25 × 10^−9^ A^−1^m) compared to that of CFO-NC (0.8 × 10^−9^ A^−1^m), but with the combined effect of Zr substitution and magnetic field applied during compaction a dramatic 435% enhancement in strain sensitivity has been observed for Co_1.2_Zr_0.2_Fe_1.6_O_4_ (4.3 × 10^−9^ A^−1^m) compared to CFO-NC (0.8 × 10^−9^ A^−1^m). Inset of Fig. 7 (lower left corner) shows the field at maximum strain sensitivity of normally and magnetically pressed samples. From the plot it is observed that slight decrease in field at (dλ/dH)_max_ is observed for magnetically compacted sample compared to normally compacted CFO. For both the normally and magnetically compacted samples, field at (dλ/dH)_max_ is observed to increase after x = 0.1 composition and this variation might be related to the larger anisotropies present in the system after x = 0.1. The magnetostriction and strain sensitivity values obtained in the present investigation are almost similar to the values reported in existing literature on magnetic annealing^11, 12^. The larger stain sensitivity (7.7 × 10^−9^ A^−1^m) reported by Wang et.al. compared to the value achieved in our study (4.3 × 10^−9^ A^−1^m) could be due to the larger magnetic fields (~2T) applied during shaping of the samples compared to the fields applied (~1T) in our study. From this result we can conclude that effect of substitution of non-magnetic ion into the spinel lattice is more compared to the effect of compaction in the presence of magnetic field in enhancing the strain sensitivity. But, in the case of magnetostriction (λ_max_) maximum effect is observed with magnetic compaction rather than the Zr^+4^ ion substitution. Therefore, Zr substituted cobalt ferrite (Co_1.2_Zr_0.2_Fe_1.6_O_4_) processed by magnetic field assisted compaction can be considered as potential material for stress sensor application.

## Methods

A series of Zr/Co co-substituted cobalt ferrite samples, with nominal compositions, Co_1+x_Zr_x_Fe_2−2x_O_4_ (where x = 0.0 to 0.4), were synthesized by conventional ceramic method using Fe_2_O_3_, ZrO_2_ and Co_3_O_4_ powders as precursors. Stoichiometric amounts of these metal oxide precursor powders were ball milled for 3 hrs and calcined twice at 1000 °C for 24 hours to enable the phase formation. To reduce the particle size calcined powders were subsequently ball milled for 5 hrs. These powders were pressed into pellets using uniaxial compaction at an applied pressure of ~150 MPa, in the presence of magnetic field (~1 Tesla) followed by sintering at 1300 °C for 12 hours.

Crystal structure of the sintered ferrites was analyzed by a Bruker D8 Advance X-ray Diffractometer (XRD) in θ–2θ geometry using Cu Kα radiation in the 2θ range between 15°–75°. The microstructural analysis of the sintered pellets was performed using a HITACHI S-3400N Scanning Electron Microscope (SEM). Room temperature magnetic properties of the samples were studied using Quantum design physical property measurement system (PPMS) at an applied magnetic field up to ~4000 kA/m. Torque measurements were performed using a torque magnetometer. In such measurement, the amplitude of the applied magnetic field was kept constant, whereas the direction was changed from 0° to 360° (clockwise) then back from 360° to 0° (counterclockwise) with a step of 5°. Magnetostrictive strain (λ) as a function of applied magnetic field (H) was measured at room temperature using the standard strain gauge method.

### Data availability

The datasets generated during and/or analyzed during the current study are available from the corresponding author on request.

## Conclusions

Structural, magnetic and magnetoelastic properties of magnetically compacted Co_1+x_Zr_x_Fe_2−2x_O_4_ (0 ≤ × ≤ 0.2) samples have been studied and the results were compared with the normal pressed samples. Parallel and perpendicular magnetization data of magnetically pressed Zr-substituted cobalt ferrite samples indicated an induced easy direction of magnetization and that was developed at nearly 90° to the direction of compaction, which introduced some uniaxial anisotropy in the samples along the same direction. Magnetostriction and strain sensitivity are increased by ~111% and 435%, respectively, for magnetically pressed Co_1.2_Zr_0.2_Fe_1.6_O_4_ compared to CFO-NC. Substitution of non-magnetic ions, having preference for tetrahedral coordination, is more effective in increasing the magnetostriction of CFO. Magnetic field assisted compaction strongly influences the strain sensitivity of substituted CFO samples. The present study indicates that magnetic compaction could be an alternate and effective method to magnetic annealing for improving the magnetoelastic properties of CFO.

## Electronic supplementary material

Supplementary Information
