# Supplementary material for: Colossal piezomagnetic response in magnetically pressed Zr+4 substituted cobalt ferrites
Source: Sci Rep. 2017 Aug 11;7:7935. doi: 10.1038/s41598-017-08160-1 (PMC5554159; doi:10.1038/s41598-017-08160-1)
Supplement: Supplementary file 1 — Supplementary Information [file 41598_2017_8160_MOESM1_ESM.pdf]

Colossal Piezomagnetic Response in Magnetically Pressed Zr^{+4} Substituted Cobalt Ferrites

Monaji Vinitha Reddy[1], Abdellah Lisfi[2], Sabin Pokharel[2] and Dibakar Das[1,*]

[1]School of Engineering Sciences and Technology, University of Hyderabad, Hyderabad, AP 500046

[2] Department of Physics, Morgan State University, Baltimore, MD 21251, USA

[*] Corresponding author email: ddse@uohyd.ernet.in

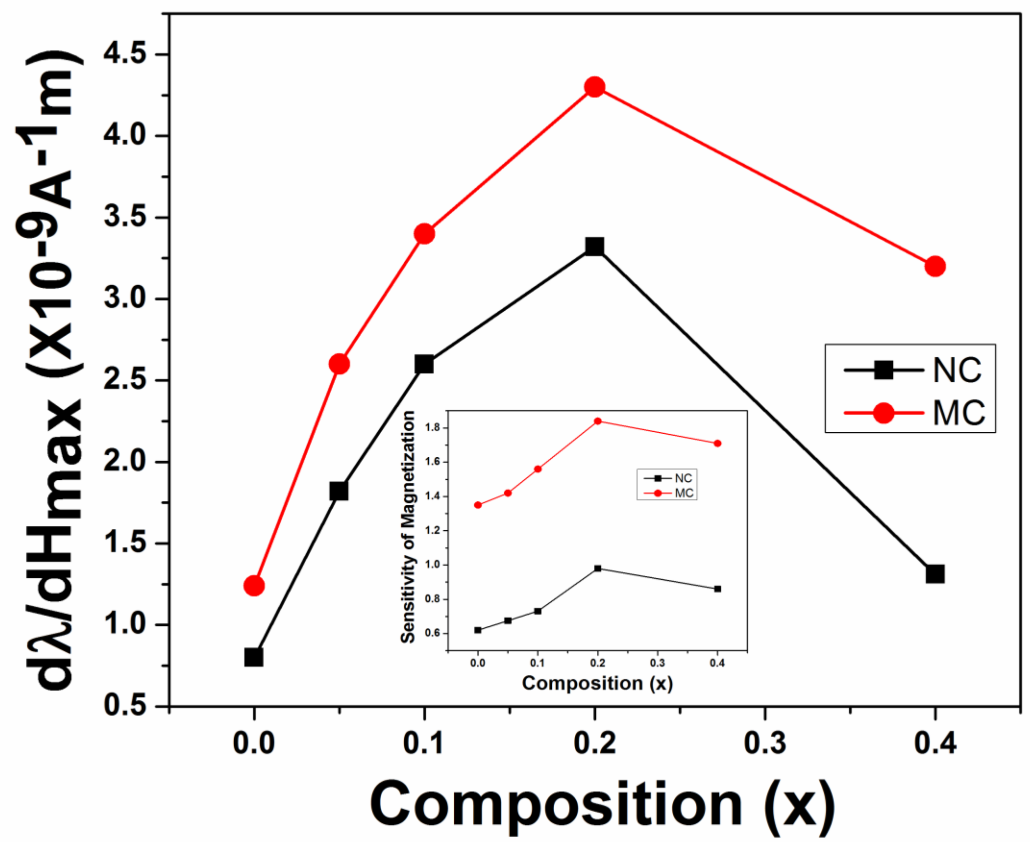

Supplementary Fig. S1: Variation of maximum strain derivative ($(d\lambda/dH)_{max}$) and sensitivity of magnetization (Inset) as a function of composition
